# Local Myeloid-Derived Suppressor Cells Impair Progression of Experimental Autoimmune Uveitis by Alleviating Oxidative Stress and Inflammation

**DOI:** 10.1167/iovs.64.13.39

**Published:** 2023-10-25

**Authors:** Jae-Young Lee, Sueon Kim, Hyun-Jung Sohn, Chang-Hyun Kim, Tai-Gyu Kim, Hyun Soo Lee

**Affiliations:** 1 Department of Ophthalmology, Eunpyeong St. Mary's Hospital, College of Medicine, The Catholic University of Korea, Seoul, Republic of Korea; 2 ViGenCell Inc., Seoul, Republic of Korea; 3 Catholic Hematopoietic Stem Cell Bank, College of Medicine, The Catholic University of Korea, Seoul, Republic of Korea; 4 Department of Molecular Medicine, The Scripps Research Institute, La Jolla, California, United States

**Keywords:** antioxidative stress, experimental autoimmune uveitis (EAU), myeloid-derived suppressor cells (MDSCs), regulatory T cells (Treg)

## Abstract

**Purpose:**

To evaluate the immune regulatory effect of human cord blood myeloid-derived suppressor cells (MDSCs) in experimental autoimmune uveitis (EAU) models.

**Methods:**

MDSCs (1 × 10^6^) or PBS were injected into established C57BL/6 EAU mice via the subconjunctival route on days 0 and 7. The severity of intraocular inflammation was evaluated for up to 3 weeks. Tissue injury and inflammation were analyzed using immunolabelled staining, real-time PCR, and ELISA. In addition, immune cells in draining lymph nodes (LNs) were quantified using flow cytometry.

**Results:**

After 21 days, the clinical scores and histopathological grades of EAU were lower in the MDSCs group compared with the PBS group. Local administration of MDSCs suppressed the oxidative stress and the expression of TNF-α and IL-1β in the retinal tissues. In addition, it inhibited the activation of pathogenic T helper 1 (Th1) and Th17 cells in draining LNs. MDSCs increased the frequency of CD25^+^ Foxp3^+^ regulatory T cells and the mRNA expression of IL-10, as an immune modulator.

**Conclusions:**

MDSCs suppressed inflammation and oxidative stress in the retina and inhibited pathogenic T cells in the LNs in EAU. Therefore, ocular administration of MDSCs has therapeutic potential for uveitis.

Uveitis is an inflammatory disease of the uvea and surrounding tissues; it is one of the major causes of blindness.^1^^,^^2^ Noninfectious uveitis represents the majority of uveitis associated with autoimmune diseases, such as juvenile idiopathic arthritis, inflammatory bowel disease, Vogt–Koyanagi–Harada, and Behcet's disease.^3^^,^^4^ The mechanisms underlying ocular inflammation in uveitis remain unclear; therefore, cases of uveitis are treated with nonspecific and sometimes systemic therapeutics, such as steroids and immunosuppressants, including cyclosporine, tacrolimus, and methotrexate.^5^^–^^7^ However, long-term use of these drugs often has adverse systemic or ocular complications, including hyperglycemia, hypertension, kidney failure, glaucoma, secondary bacterial infections, and cataracts. Therefore, more specific and less adverse therapeutics based on immunological mechanisms are necessary for overcoming drug-related complications.^8^

The pathological lesions of clinical uveitis are reflected in experimental autoimmune uveitis (EAU) animal models through immunization with retinal proteins, such as interphotoreceptor retinoid-binding protein (IRBP).^9^^,^^10^ Understanding the immunopathological mechanisms in EAU models will help to develop therapeutic targets for uveitis.^11^ EAU is characterized by severe inflammation of the retina and uvea; it could be divided into early and amplified phases.^12^ CD4^+^ T-cell–mediated adaptive immune responses are associated with the amplified phase of EAU; in addition, innate immune responses mediated by macrophages, dendritic cells, and retinal microglia are important for early phase of uveitis.^13^^,^^14^ In the early phase, pathogenic peroxynitrite of the photoreceptor induces retinal mitochondrial oxidative stress; in addition, it is a trigger for innate immunity-mediated inflammation.^15^^,^^16^ At 5 days after immunization of EAU, significant upregulation of inflammatory cytokines, such as TNF-α, inducible nitric oxide synthase (iNOS), IFN-γ, and IL-1, is associated with the induction of oxidative stress in the retina.^17^ M1 classical macrophages, which can be activated by IFN-γ secreted from infiltrated T cells, produce TNF-α and IL-6. This process leads to lipid peroxidation and surrounding tissue injury, and contributes to the pathological initiation of EAU.^18^^,^^19^ The dominant adaptive immune responses, including pathogenic T helper 1 (Th1) and Th17 cells, play a critical role in the progression of ocular inflammation in the amplified phase of the EAU model.^20^^,^^21^

Myeloid-derived suppressor cells (MDSCs) exhibit an immune suppressive function in tumor environments and in immune-related inflammation.^22^ MDSCs suppress CD8^+^ T cells in tumors, through iNOS and arginase enzymes.^23^^,^^24^ The immunosuppressive function of MDSCs in various inflammatory diseases has gathered wide attention.^23^^–^^25^ The therapeutic administration of MDSCs alleviates TNF-α–mediated inflammation and inhibits activation of pathogenic Th1 and Th17 cells, through the induction of regulatory T (Treg) cells and the upregulation of immune regulatory mediators, such as IL-10 and TGF-β.^26^^–^^28^

We investigated the suppressive functions of MDSCs in intraocular inflammation in an IRBP-mediated EAU C57BL/6 mice, which naturally exhibit Th1 and Th17 responses.^29^ Local administration of MDSCs significantly alleviated intraocular inflammation and the clinical severity of EAU by inhibiting oxidative stress and inflammatory tissue injury, possibly mediated by the induction of Treg cells and IL-10.

## Methods

### Culture of MDSCs

Our experimental procedures using human cord blood derivatives, including MDSCs, were performed under guidelines endorsed by Korea National Institute for bioethics policy (IRB no. P01-202010-31-008). MDSCs were prepared according to a previously described our method (see Supplementary Methods).^30^

### Flow Cytometric Analysis of MDSCs

Isolated MDSCs were stained with anti-CD16/CD32 (Cat# 564219, BD Biosciences, San Jose, CA, USA) for Fc receptor blocking on ice and then incubated with the anti-human antibodies. The expression of MDSCs was evaluated by monoclonal antibodies specific to markers, including CD33 FITC (Cat# 11-0339-042, Invitrogen, Waltham, MA, USA), CD11b PE (Cat# 12-0118-42, Invitrogen), and CD14 PE-Cy7 (Cat# 25-0149-42, Invitrogen). For intracellular staining of iNOS FITC (Cat# SC-7271, FITC, Santa Cruz Biotechnology, Dallas, TX, USA), IDO PE (Cat# IC6030P, R&D Systems, Bio-Techne, Minneapolis, MN, USA), and ARG1 PerCP (Cat# IC8026C, R&D Systems). MDSCs were incubated for fixed and permeabilized using BD Cytofix buffer and BD Cytoperm buffer (Cat# 554714, BD Biosciences). All samples were acquired with BD Lyric (BD Biosciences) and then analyzed with FlowJo software (v10.8.1, FlowJo LLC, Ashland, OR, USA).

### Animals

Six- to 8-week-old female C57BL/6 mice from Orient Bio Inc (Seongnam, Kyonggi-do, Korea) were housed under specific pathogen-free condition. In this study, animal experiments were conducted according to the guidelines in the Catholic Institutional Animals in Ophthalmic and the ARVO Statement for the Use of Animals in Ophthalmic and Vision Research (IACUC Approval no. EPS-MH-2020-1701-FA). Anesthesia was induced by intraperitoneal injection of ketamine (120 mg/kg) and xylazine (20 mg/kg).

### Induction of EAU in Mice

EAU induction in mice by injection of IRBP was performed based on the previously described methods.^9^ Briefly, the mice were immunized with 200 µg of emulsion containing 100 mg/70 mL of *Mycobacterium tuberculosis* Des H37Ra (BD, Dickinson and Company, Franklin Lakes, NJ, USA) and IRBP peptide (300 µg; residues, 21-13301, 1-20 GPTHLFQPSLVLDMAKVLLD; PEPTRON, Daejeon, Korea) in Freund's Adjuvant (Cat# F5881, Sigma-Aldrich, St. Louis, MO, USA), administrated in each foot pad with the intraperitoneal injection of pertussis toxin (0.7 µg) as an adjuvant. Clinical EAU scores were observed with microscopic examination at 1, 2, and 3 weeks after MDSCs or vehicle (PBS) treatment on a scale of 0 (no disease) to 4 (severe disease) in a blinded manner, using the criteria based on description of Bansal et al.^31^^,^^32^

### Injection With Cord Blood MDSCs

Human cord blood MDSCs (ViGenCell Institute and the Catholic Hematopoietic Stem Cell Bank, an affiliation of the College of Medicine, The Catholic University of Korea) was obtained to investigate the therapeutic effect in EAU. The administration of MDSCs was injected via subconjunctival injection on the first day and day 7 in the IRPB-immunized mice, which was suspended in PBS at 1 × 10^6^/10 µL volume. The vehicle (PBS) group mice were subconjunctival injected with the same volume of PBS as the MDSCs group. Conjunctival injections can occasionally lead to local irritation, hemorrhages, necrosis, and granuloma at the injection site.^33^^,^^34^ In our experiment, the only adverse reaction observed at MDSCs administration was conjunctival bleeding on one eye, which resolved within 1 week.

### Histological Hematoxylin and Eosin Staining

The slides of cryosectioned eyeballs were stained by hematoxylin and eosin. Photographs were obtained with DMI 5000B microscope (Leica, Wetzlar, Germany) in a blinded fashion at 200× magnification. To assess the pathological score of the retina was evaluated (scale of 0–4 scores) using a blinded manner based on the previously proposed criteria.^35^^,^^36^

### Immunolabelled Tissue Staining

Tissue-Tek O.C.T compound (Cat# 4583, Sakura Finetek, Torrance, CA, USA) embedded mouse eyes were sectioned and permeabilized (0.1% Triton X100 in PBS) and blocked by BSA buffer (5% BSA, Sigma-Aldrich) before incubation with primary antibody (diluted 1:200, 8OhdG antibody, Cat# ab48508, Abcam, Cambridge, MA, USA) for oxidative stress assay. Then incubation at 37°C with Alexa Fluor 594 Goat anti-mouse secondary antibody (diluted 1:400, Cat# ab150116, Abcam). Retinal apoptosis was measured using the instructions of the manufacturer's In Situ Cell Death Detection Kit (Roche Diagnostics, Indianapolis, IN, USA), as previously described.^37^ All immunolabeled slide tissue was mounted using DAPI mounting medium (Vector Laboratories, Burlingame, CA, USA), then were examined using Axiovert 200 fluorescence microscope (Carl Zeiss, Overkochen, Germany). A immunohistochemistry kit (Cat# 64264, Abcam) was applied to stain the samples for cytokine expression. Primary antibodies were stained with anti-mouse TNF-α antibody (1:200, Cat# sc-52746, Santa Cruz Biotechnology) and anti-mouse IL-1β antibody (1:200, Cat# sc-52012, Santa Cruz Biotechnology). The counterstaining was with hematoxylin and then the tissue slides were mounted with a VectaMount Permanent Mounting Medium (Vector Laboratories). Images were obtained by DMI 5000B light microscope (Leica). The quantification of field image was performed by calculating the percentage of cytokine area or the number of positive cell counts using the software Image J, as previously described.^38^ Each group consisted of three or four animals, and all quantification of retinal images was evaluated in a* *blinded manner as the mean of three or four selected images for each group.

### ELISA for Cytokine Expression

After euthanasia using a CO_2_ chamber at 3 weeks after immunization, the serum in whole blood by cardiac puncture was collected for mouse IFN-γ (Cat# 430807, BioLegend, San Diego, CA, USA), IL-17 (Cat# 432507, BioLegend), TNF-α (Cat# 430907, BioLegend), and IL-1β (Cat# BMS6002, Invitrogen) analyses. ELISA kits were analyzed from accordance with the directions provided by the manufacturer. The samples were then analyzed with an ELISA microtiter plate auto reader at 450 nm (Molecular Devices, San Jose, CA, USA).

### Real-Time PCR

Total RNA of the retina and choroid were isolated using Trizol (Invitrogen) and RNeasy Mini (Cat# 74106, Qiagen, Germantown, MD, USA) and three or four samples were pooled randomly within their respective groups. First-strand cDNA was synthesized with PCR Amplified cDNA (Cat# 12574026, Invitrogen), and a quantitative real-time PCR was detected that FAM dye-labeled predesigned primers (IFN-γ: Mm01168134_m1, IL-17: Mm00439618_m1, IL-10: Hs00961622_m1, glyceraldehyde 3-phosphate dehydrogenase: Mm99999915_g1, ThermoFisher, Rockford, IL, USA). For each reaction, the housekeeping gene was used as an internal control. All sample data were measured by the comparative threshold cycle method using the Quantity One 1-D analysis software (Bio-Rad, Hercules, CA, USA), and the relative expression was expressed as fold changes compared with a gene between positive samples and internal naive samples.

### Flow Cytometry Analysis

Draining lymph nodes (LNs) were separated into single cells on postimmunization day 21 through a 70 µm cell strainer, and 5 × 10^5^ single cells, pretreated with stimulation Cocktail (plus protein transport inhibitors, Cat# 00-4975-93, eBioscience) for 6 hours, were dispensed into each tube along with intracellular/intranuclear staining fixation solution in 0.5% BSA. The antibodies staining was incubated with anti–IFN-gamma PE (Cat# 505807, BioLegend), anti–IL-17 Alexa647 (Cat# 506912, BioLegend), anti-CD4 Alexa488 (Cat# 100529, BioLegend), anti-CD25 PE (Cat# 12-0251-82, ThermoFisher), and anti-Foxp3 PE-Cy7 (Cat# 25-5773-80, eBioscience, San Diego, CA, USA). The stained cells were acquired on BD FACSMelody (BD Biosciences) and analyzed with FlowJo Software 10.5.3 (FlowJo LLC).

### Statistical Analysis

Data were demonstrated statistical significance by Prism version 5 (GraphPad Software, La Jolla, CA, USA) and were expressed as mean ± SD. Differences between groups were classified by the Student *t*-test or one-way ANOVA post hoc Tukey's test. Kaplan Meier curves were analyzed by log-rank test. A *P* value of less than 0.05 was considered statistically significant.

## Results

### Characterization of CB-MDSCs

**Figure 1.**
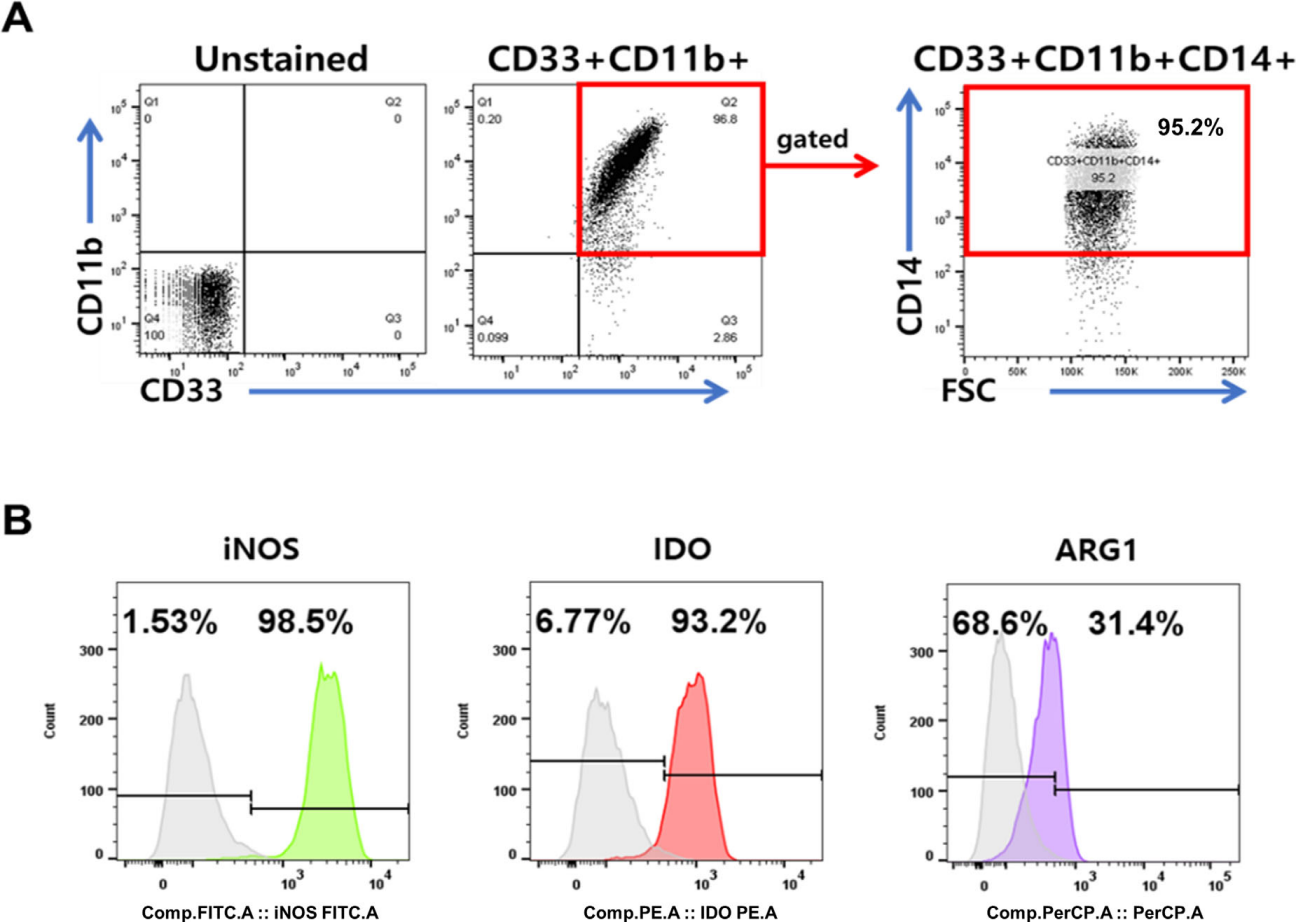
Phenotypic and functional characteristics of human umbilical cord blood-derived MDSCs. (A) Flow cytometry was used to analyze MDSCs stained with individual MDSC surface marker antibodies. (B) The expression of immune suppressive molecules in MDSCs, as indicated by staining with FITC anti-iNOS2 antibody, PE anti-IDO antibody, and PerCP-Cy5.5 anti-ARG1 antibody.

CB-MDSCs were cultured as described previously.^27^ Flow cytometry analysis confirmed the presence of the MDSCs surface markers, including CD33, CD11b, and CD14 in the CB-MDSC population (Fig. 1A). Intracellular staining confirmed the expression of well-characterized MDSC immunosuppressive molecules, such as iNOS, IDO, and ARG1 (Fig. 1B).

### Amelioration of the Clinical Score and Histological Grade of EAU After MDSC Administration

**Figure 2.**
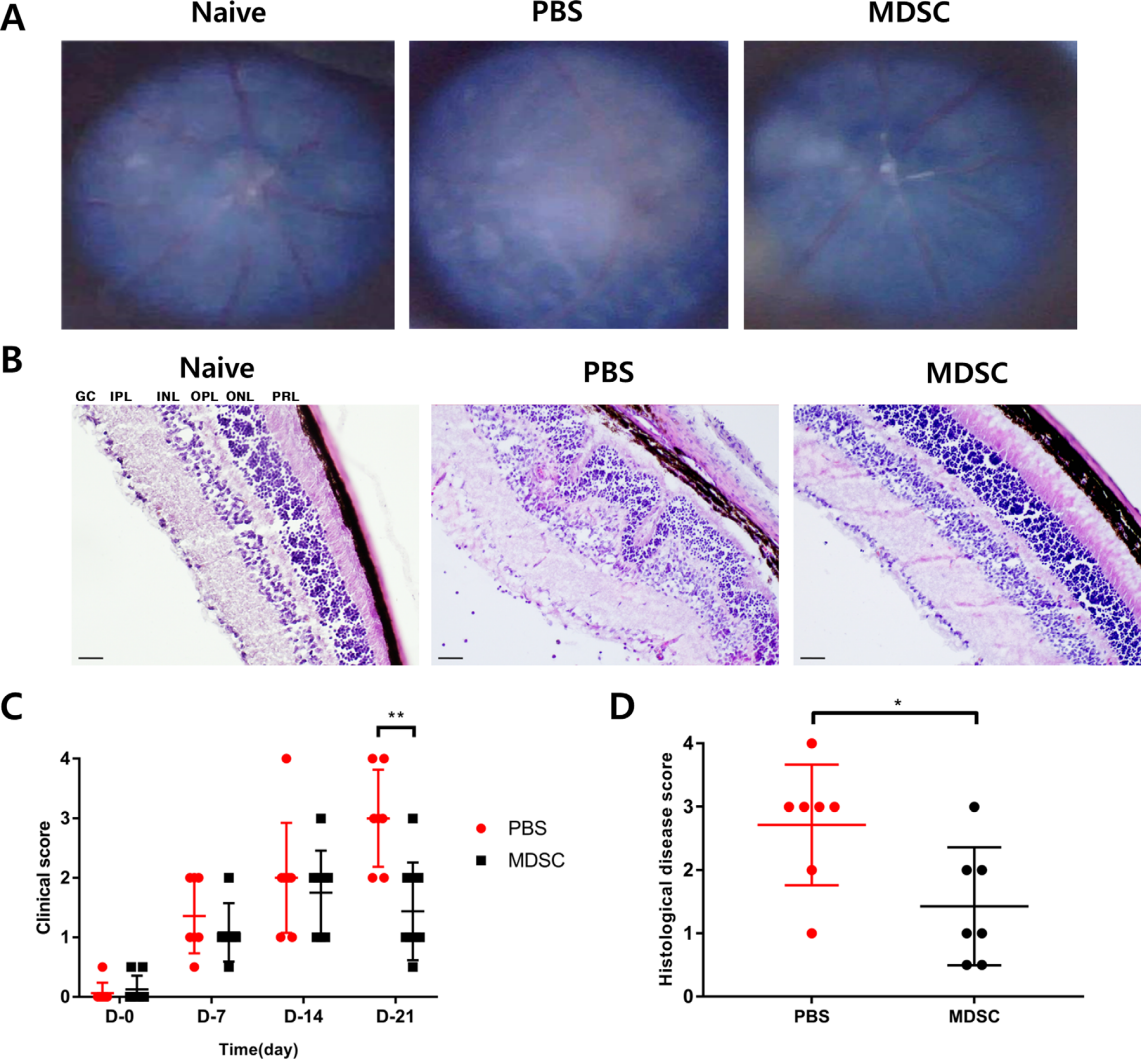
Clinical scoring and histopathological grading of the retina following the administration of MDSCs in EAU. (A) Fundus images of mice showing representative photos in each group. (B) Histologic representative images indicating retinal tissues stained with hematoxylin and eosin in each group. Scale bars, 50 µm. GCL, ganglion cell layer; IPL, inner plexiform layer; INL, inner nuclear layer; OPL, outer plexiform layer; ONL, outer nuclear layer; PRL, photoreceptor layer. (C) Locally injected MDSC group (*n* = 8) displayed significantly less intraocular inflammatory scores than the PBS group (*n* = 7) for 21 days (**P = 0.0027). (D) The histopathological grading of EAU showed a significant decrease in the MDSC group (*n* = 7) compared with that in the PBS group (*n* = 8) (*P = 0.0253). Data are represented as mean ± SD of three independent experiments.

Using the EAU model, we confirmed the therapeutic effects of MDSC administration during the early stages of uveitis. MDSCs (1 × 10^6^/10 µL) or PBS as a vehicle control (10 µL) were administered via subconjunctival injection on days 0 and 7 after immunization in each group. Intraocular inflammation was evaluated for up to 3 weeks after IRBP immunization (Fig. 2A). The clinical EAU scores were significantly different between the MDSC- and PBS-administered groups at 3 weeks (Fig. 2C) (*P* < 0.01). The PBS group exhibited apparent pathological changes consistent with uveitis, such as diffuse retinal detachment, subretinal bleeding, and retinal folding at 3 weeks, compared with the MDSCs group (Figs. 2A, 2B). The histological disease grades in the MDSCs group were significantly lower than those in the PBS group (Fig. 2D) (*P* < 0.05). Subconjunctival injection of MDSCs significantly alleviated the severity of uveitis with decreased clinical scores and histological grades of EAU.

### Decreased Activation of Pathogenic Th Cells With MDSCs in Uveitis

**Figure 3.**
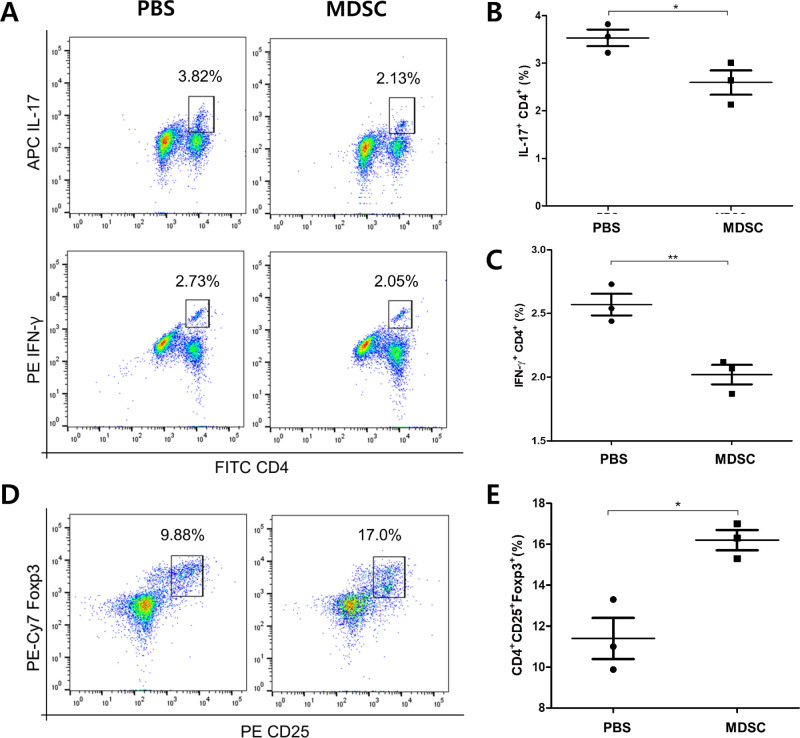
Frequencies of pathogenic effector Th1 and Th17 cells and Treg cells in draining LNs in EAU. (A) Representative flow cytometry plots showing populations expressing IFN-γ or IL-17 cells after CD4^+^ gating. (B) Quantitative analysis of IL-17^+^ CD4^+^ cell populations (*n* = 3). (C) Quantitative analysis of IFN-γ CD4^+^ cell populations (*n* = 3). Comparison between MDSCs and PBS groups showed that both Th1 and Th17 cells presented decreased frequencies in MDSCs group. (D) Representative flow cytometry plots showing the population of CD25^+^ Foxp3^+^ cells after CD4^+^ gating. (E) Quantitative analysis graph of CD4^+^ CD25^+^ Foxp3^+^ cells (Treg) (*n* = 3). MDSC group had an increased ratio of Treg cells when compared with that in the PBS group. Representative flow cytometric data from three independent trials with pooled cells from more than three mice per group. *P < 0.05; **P < 0.01.

To determine whether the administration of MDSCs could be involved in T-cell activation in EAU, the population of CD4^+^ T cells expressing IL-17 or IFN-γ in draining LNs at 3 weeks was measured using flow cytometric analysis. MDSC treatment decreased the frequencies of IL-17^+^ CD4^+^ T cells and IFN-γ^+^ CD4^+^ T cells compared with that in the PBS group (Figs. 3A–3C) (*P < 0.05 and **P < 0.01, respectively). MDSCs administration reduced the differentiation and proliferation of Th17 and Th1 cells, the main pathogenic immune responses in uveitis. In addition, the frequency of CD4^+^ CD25^+^ Foxp3^+^ Tregs was significantly increased in the MDSCs group compared with that in the control group (Figs. 3D, 3E) (*P < 0.05). Moreover, the population of CD3^+^ CD127^+^ Tregs in draining LN tissues were also significantly higher in the MDSCs group, compared with the PBS group (Supplementary Fig. S1) (***P < 0.01).

### Prevention of Inflammatory Tissue Injury by MDSC Administration in Uveitis

**Figure 4.**
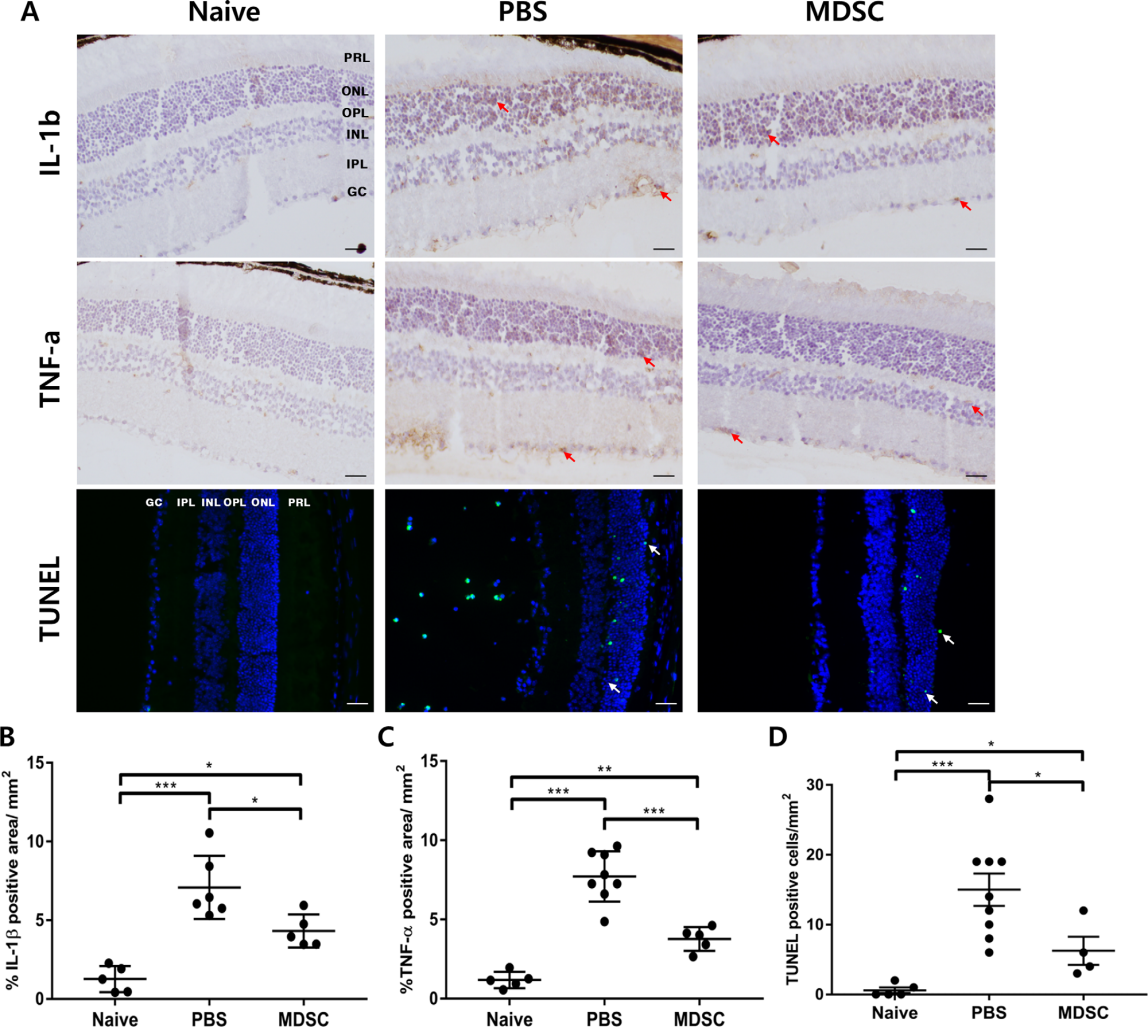
Immunohistochemical staining and TUNEL assay of retinal cross-section for evaluating cytokine expression and cellular apoptosis in EAU. (A) Cross-sectional images of the mouse retinas showing immunohistochemical staining using IL-β and TNF-α, and the staining of TUNEL (*white arrow*; *green staining*) assay. Scale bar, 50 µm. GCL, ganglion cell layer; IPL, inner plexiform layer; INL, inner nuclear layer; OPL, outer plexiform layer; ONL, outer nuclear layer; PRL, photoreceptor layer. (B–D) Quantification graphs for each cytokine expression and TUNEL-positive apoptotic cells. In the MDSCs group, the expression of the cytokines, IL-1β and TNF-α were significantly decreased in the retina, compared with that in the PBS group. In addition, MDSC group showed decreased cellular apoptosis (green) in the retinal cross-section, compared with that in the PBS group. Different sections from four or more independent mice were randomly selected for analyzing blinded samples, and the data were represented as mean ± SD of three independent experiments. *P < 0.05; **P < 0.01; ***P < 0.001.

The levels of IL-1β and TNF-α expression in retinal tissues were significantly lower in the MDSCs group compared with that in the PBS group (Figs. 4A–4C) (*P* < 0.05 and *P* < 0.001, respectively). Extracellular adenosine secreted from MDSCs inhibits the production of TNF-α by activating A2a/A2b receptors in inflammatory cells. Similarly, the levels of TNF-α expression in draining LNs were significantly lower in the MDSCs group compared with the PBS group (Supplementary Fig. S2A) (**P < 0.01). In addition, MDSCs release IL-1 receptor antagonist (IL-1RA) and arginase, which could suppress T-cell activity through competitive inhibition of the inflammatory IL-1 receptor and inhibition of T-cell proliferation by l-arginine depletion, respectively.^39^^–^^41^ The PBS group showed a significant increase in apoptotic cells at the damaged tissue areas; here, the inflammatory cytokines were upregulated compared with the MDSCs group (Fig. 4D) (*P* < 0.05). Moreover, MDSCs group showed increased levels of IL-10 expression compared with the PBS group in draining LN (Supplementary Fig. S2B) (**P < 0.01). Therefore, local MDSCs administration can prevent tissue damage by suppressing inflammatory cytokine production and cellular apoptosis in patients with EAU.

### Decreased Oxidative Stress With MDSC Administration in Uveitis

**Figure 5.**
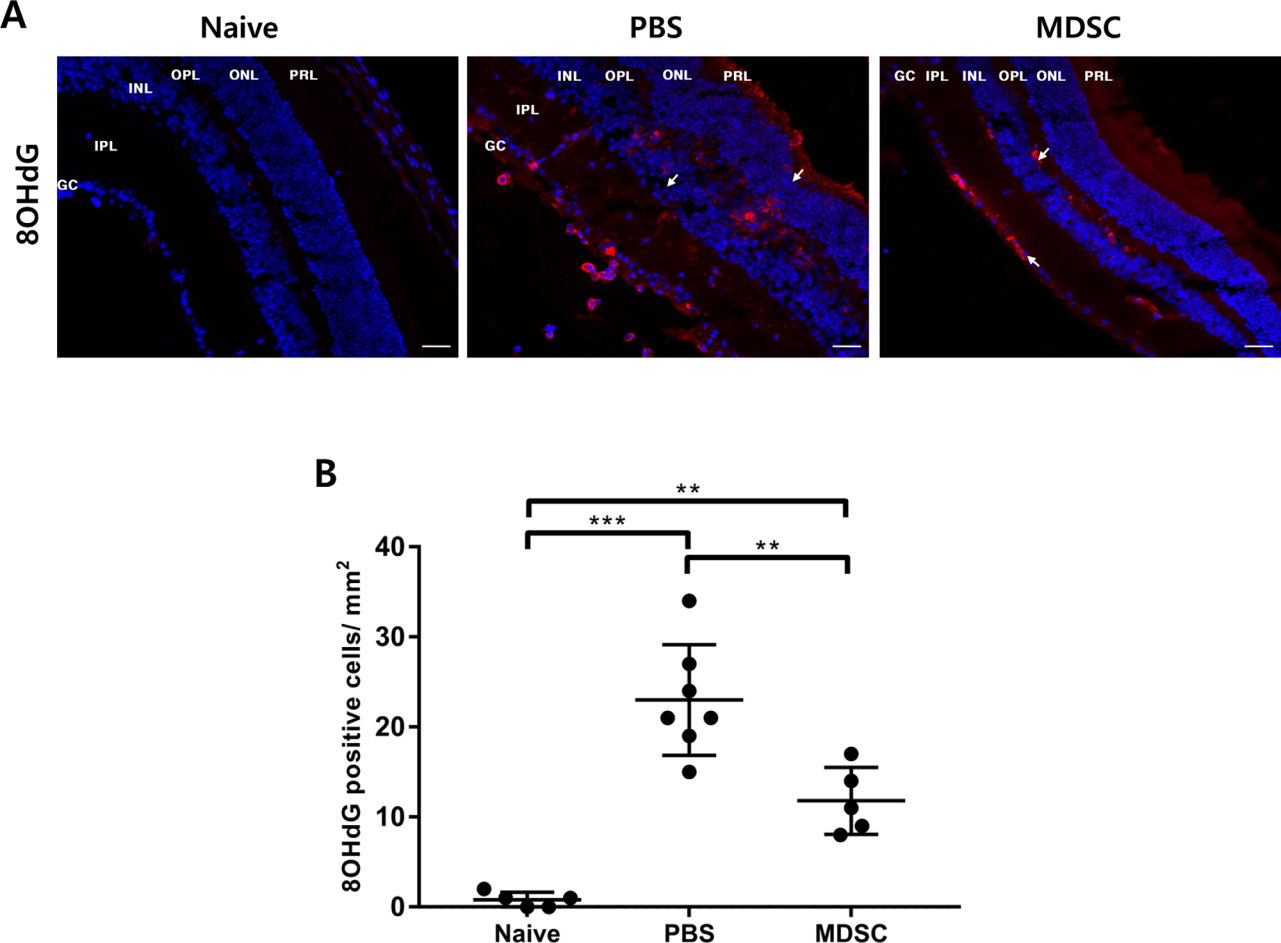
Assessment of oxidative stress in the retina at 3 weeks after IRBP immunization. (A) Representative 8-hydroxy-2-deoxyguanosine labeling assay images of the retinal histologic section in each group (*white arrow*, 8-OHdG [*red*]; counterstained with DAPI [*blue*]). Scale bar, 50 µm. GCL, ganglion cell layer; IPL, inner plexiform layer; INL, inner nuclear layer; OPL, outer plexiform layer; ONL, outer nuclear layer; PRL, photoreceptor layer. (B) Quantitative analysis of 8-OHdG positive cells. MDSC group showed suppressed oxidative stress on the retina compared with that in the PBS group. Different sections from four or more independent mice were randomly selected for analyzing blinded samples, and data were represented as mean ± SD of three independent experiments. **P < 0.01; ***P < 0.001.

Oxidative stress is increased in autoimmune diseases, such as uveitis and rheumatoid diseases; this factor contributes to inflammatory immune responses.^21^^,^^42^ Oxidative stress a key pathogenic mechanism underlying innate immunity-mediated inflammation in retinal pigment epithelial cells in EAU.^12^^,^^21^ Immunohistochemical staining for the 8OHdG marker in retinal tissues was performed to confirm oxidative stress at 3 weeks (Fig. 5A). The MDSCs group showed significantly suppressed oxidative stress, compared with the PBS group (Fig. 5B) (*P* < 0.01). Therefore, local administration of MDSCs can suppress both inflammatory responses and oxidative stress in the tissues of EAU mice.

### MDSCs-Mediated Suppression of Local and Systemic Expression of Inflammatory Cytokines in Uveitis

**Figure 6.**
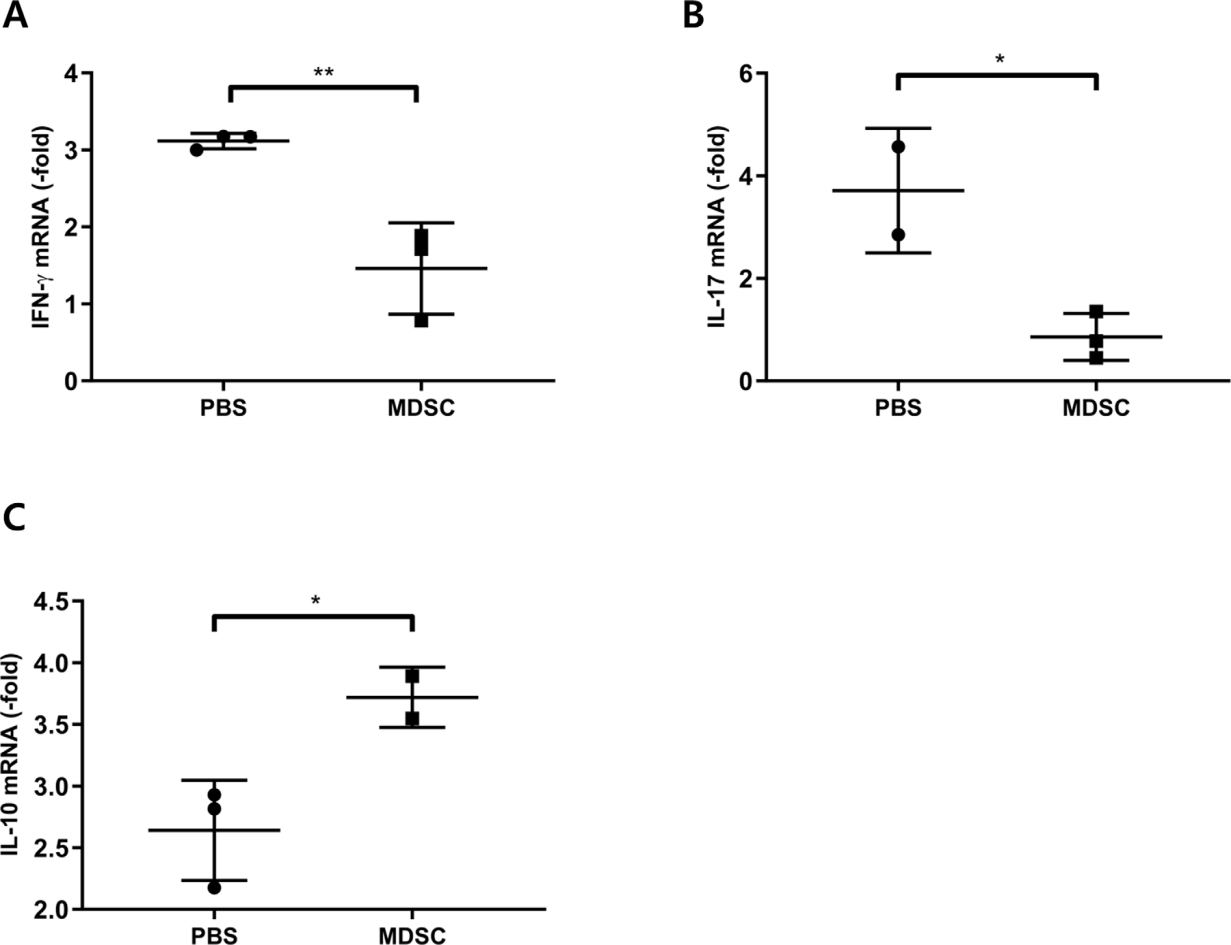
Real-time PCR analysis of the mRNA expression levels of proinflammatory and immunoregulatory genes in the retina and choroid. mRNA levels of proinflammatory (A, IFN-γ; B, IL-17) and immunoregulatory (C, IL-10) genes in the retina and choroid at 3 weeks after immunization. Local administration of MDSCs decreased mRNA expression of IFN-γ (A) and IL-17 (B) compared with that in the PBS group; however, the MDSCs group showed increased expression of IL-10 (C). Data were normalized using glyceraldehyde 3-phosphate dehydrogenase as the internal control, and relative values were expressed as the fold change of the expression in the naïve retina. Data were presented as mean ± SD of three experiments (*n* = 3; *P < 0.05; **P < 0.01).

**Figure 7.**
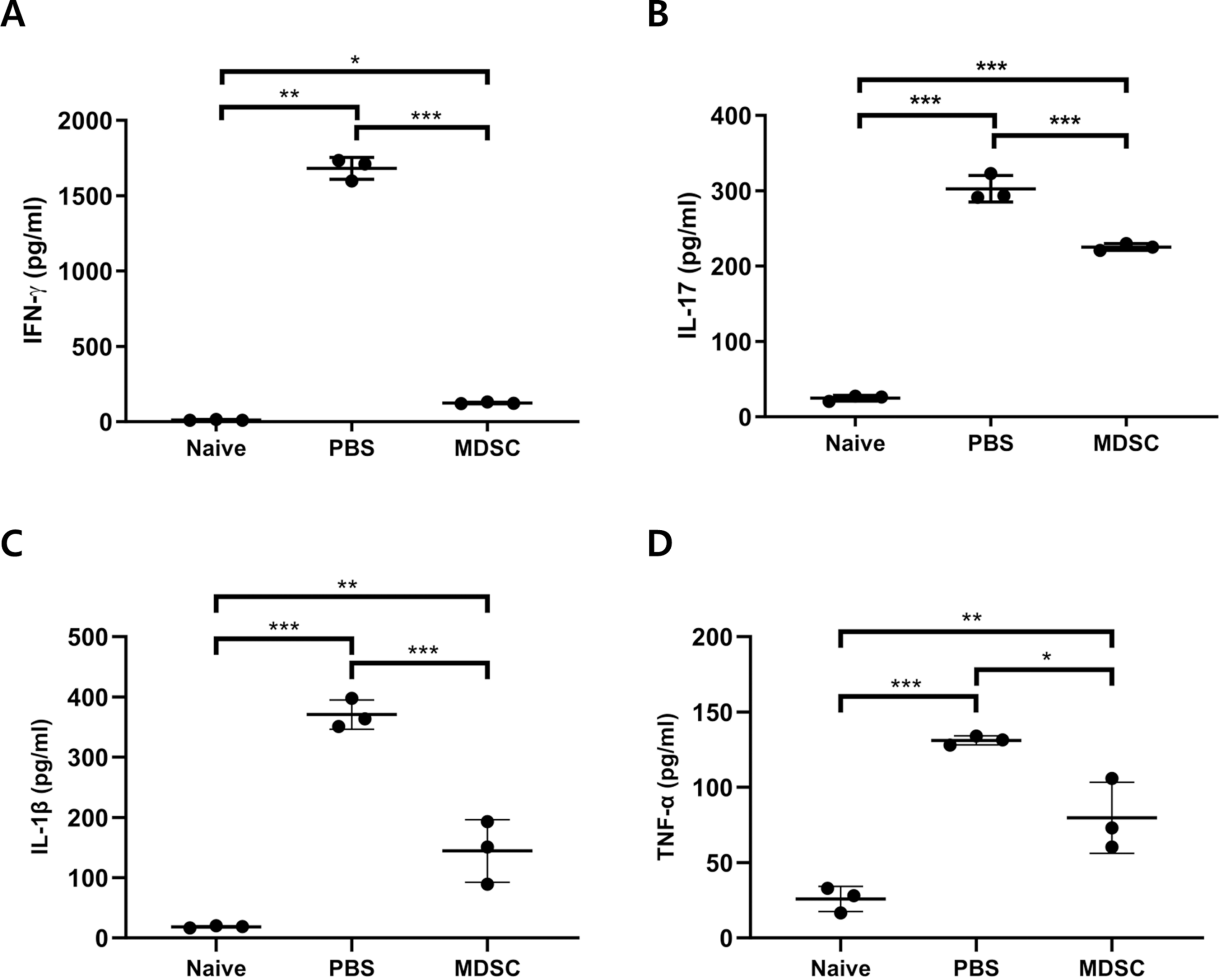
ELISA of inflammatory cytokines profile in the serum. The local administration of CB-MDSC significantly decreased production of IFN-γ (A), IL-17 (B), IL-1β (C), and TNF-α (D) in the serum, compared with those of the PBS-treated groups. The serum was quantified by ELISA on day 21, and naive groups have obviously low expression comparison with PBS-treated groups. Data are presented as mean ± SD. Comparison between the three groups was expressed using one-way ANOVA with post hoc paired Tukey's test (*n* = 5; *P < 0.05; **P < 0.01; and ***P < 0.001).

The mRNA expression levels of inflammation-related genes were evaluated in the retina and choroid at 3 weeks; expression of the inflammatory mediators, IFN-γ and IL-17, was significantly increased in the PBS group compared with that in the MDSCs group (Figs. 6A, 6B) (*P* < 0.01 and *P* < 0.05, respectively). The mRNA expression of IL-10, an immune modulator, was significantly increased (Fig. 6C) (*P* < 0.05), whereas the serum levels of both IFN-γ and IL-17 were significantly decreased at 3 weeks in the MDSCs group compared with the PBS group (Figs. 7A, 7B) (*P* < 0.001 and *P* < 0.01, respectively). In addition, serum levels of proinflammatory cytokines, such as IL-1β and TNF-α, were also significantly decreased in the MDSC group compared with the PBS group (Figs. 7C and 7D) (*P* < 0.001 and *P* < 0.05, respectively). Therefore, locally injected MDSCs can decrease local and systemic inflammatory responses in EAU, possibly mediated by increased IL-10 expression in ocular tissues.

## Discussion

Intraocular inflammation in uveitis affects the eyes; in addition, it could be caused by other systemic diseases, such as infections or autoimmune diseases. However, the pathogenesis of the various types of uveitis remains unclear. The major phenotypes of uveitis share similar immunopathogenesis, characterized by the activation of Th1 and Th17 cells and the progression of retinal pigment epithelial damages through mitochondrial oxidative stress.^12^^,^^43^ Oxidative stress is initiated in the mitochondria of photoreceptors, mediated by the upregulation of iNOS on day 5 after immunization in EAU.^17^^,^^44^ During oxidative stress and inflammatory responses, macrophages release metabolic products, such as antimicrobial peptides, reactive nitrogen species (NO), and reactive oxygen species, representative of the innate immune mechanism.^17^^,^^45^ The migration of activated microglia and macrophages into the outer retinal layer leads to the secretion of pathogenic factors, such as peroxynitrite and TNF-α.^16^^,^^46^ During early phase innate immune reactions, the release of proinflammatory mediators, including nitrogen oxides, IL-1β, IL-6, TNF-α, and reactive oxygen species, mediate tissue injuries^18^^,^^47^ and play a crucial role as an effector of innate immunity and an amplifier of acquired immunity.^48^ Subconjunctival-injected MDSCs alleviated the clinical severity and pathogenic T-cell responses in an EAU model through the inhibition of inflammatory response and oxidative stress in retinal tissues.

MDSCs are immunosuppressive immature myeloid cells that regulate pathogenic inflammatory immune responses and oxidative stress.^49^ MDSCs can migrate into excessive inflammatory conditions, including many tumors and autoimmune diseases.^50^^,^^51^ Previous studies reported that MDSCs, as observed in animal models of autoimmune diseases, not only contribute to disease progression, but can also mitigate inflammation in autoimmune encephalomyelitis by MDSCs administration.^52^^–^^56^ To the best of our knowledge, this study is the first to evaluate the therapeutic efficacy of locally injected exogenous MDSCs, which could minimize potential side effects of systemic administration of MDSCs, by suppressing oxidative stress and inflammation in preclinical uveitis models. The findings showed that local administration of MDSCs could suppress oxidative stress and inflammatory progression in EAU, which could contribute to the prevention of tissue injuries through the potential immune regulatory function. Previously, we showed that subconjunctival injection of MDSCs suppressed allograft rejection in corneal transplantation, even at lower doses of MDSCs, compared with that used for systemic intravenous administration.^37^ Therefore, we chose local (subconjunctival) injection for MDSCs administration in EAU.

Ocular administration of MDSCs on days 0 and 7 significantly decreased clinical scores and retinal histopathological lesions of EAU at week 3 after immunization. Adoptive transfer of MDSCs in the IRBP-induced uveitis model downregulated the TLR4-mediated innate immune response and decreased pathogenic T-cell activation.^57^ During the progression of uveitis, the blood–retinal barrier is disrupted, and immune cells infiltrate into the retina and choroid; this process is observed clinically as retinitis and choroiditis. The IFN-γ–producing Th1 and IL-17–producing Th17 cells play an important role in the progression of EAU with retinal and uveal tissue destruction.^14^^,^^58^^–^^61^ IFN-γ^+^ CD4^+^ cells and IL-17^+^ CD4^+^ cells in the draining LNs and the serum IL-17 and IFN-γ levels were lower after MDSCs administration. The serum levels of IFN-γ and IL-17 reduced with the cytokine profiles in human^62^ and rodent^63^ uveitis with various immunosuppressive treatments. MDSCs regulate alloreactive CD4^+^ T cells by impairing dendritic cell maturation during murine corneal transplantation.^37^ Depletion of Foxp3^+^ Tregs in uveitis leads to more severe uveitis during EAU.^64^^,^^65^ Foxp3^+^ Tregs are an important factor in resolving and maintaining EAU.^64^^,^^65^ MDSCs induce IL-10 secretion, leading to the expansion of Treg cells.^66^^,^^67^ MDSC-derived IL-10 has been reported to play a role in the induction of Treg cells and the inhibition of dendritic cells to suppress pathologic T-cell responses.^68^^,^^69^ MDSCs-derived other soluble factors, such as TGF-β and exosomes, also could induce suppressive capacity within the inflammatory environment.^70^^,^^71^ Locally injected MDSCs could suppress the progression of EAUs; this is mediated by an increased population of Foxp3^+^ Treg cells in draining LNs and increased IL-10 expression.

The TLR4-mediated nuclear factor-κB pathways is one of major immune-mediated inflammatory responses in uveitis; this pathway induces oxidative stress and proinflammatory cytokines, especially IL-1β and TNF-α.^12^^,^^13^^,^^72^ MDSCs regulate inflammatory responses, mediated by immune modulators such as IL-10 and TGF-β.^26^^,^^73^^,^^74^ Increased expression of IL-1β under inflammatory environments induces the attraction of MDSCs and increases the levels of IL-10 and Arg-1 in MDSCs.^56^^,^^75^^–^^78^ The levels of the inflammatory cytokines, TNF-α and IL-1β, are increased in the retinal and choroidal tissues in EAU. Previous studies have reported that the majority of infiltrated cells within the retina after uveitis inflammation were F4/80^+^ macrophages, which could produce proinflammatory cytokines, such as IL-1β and TNF-α, and contribute to uveitis-associated inflammation.^79^^,^^80^ MDSCs administration suppressed the expression of inflammatory cytokines, possibly mediated by increased IL-10 levels in EAU.

MDSCs administration suppressed oxidative stress and cellular apoptosis in EAU cells. Interestingly, the oxidative stress marker, 8OHdG, was most expressed in the ganglion cell layer, inner nuclear layer, and outer nuclear layer, which increased the number of apoptotic cells in the TUNEL assay. Therefore, oxidative stress is associated with increased apoptotic cell death in EAU, in correspondence with earlier reports.^13^^,^^81^ TNF-α production during the early phase of uveitis is associated with the mitochondrial oxidative stress of retinal cells.^12^^,^^16^ Anti–TNF-α agents for uveitis, such as infliximab and adalimumab, are increasingly analyzed in clinical studies.^12^^,^^13^ Therefore, MDSCs could control the progression and severity of uveitis through modulating innate and acquired immune responses in EAU.

In clinical applications, cellular therapies offer benefits while also inducing side effects depending on the route of administration. Local delivery could be more efficient, avoiding comprehensive side effects, compared with systemic delivery.^82^^,^^83^ Subconjunctival delivery of MDSCs is a stable and efficient administration route to overcome the problems associated with systemic injection, even at a dose lower than that used for MDSCs systemic administration.^37^ Therefore, the results support evaluating optimal MDSC application in future clinical trials. Systematically evaluating different dosages, cellular distributions, and other immunomodulatory activities of MDSCs in future experiments is important. Our study, based on previous established uveitis researches,^84^^–^^86^ involved the sacrifice day set at 21 days, when we observed significant differences in clinical scores and histopathological scores between PBS control and MDSCs groups to evaluate the immune regulation of exogenous MDSCs treatment. Furthermore, we will further explore the long-term and therapeutic efficacy of MDSCs after uveitis establishment in future. MDSCs present low HLA expression, which lead to successful immunosuppressive effects by avoiding HLA mismatch–related rejection after MDSCs transplantation.^29^^,^^87^^,^^88^ However, it should be considered that HLA mismatches in clinical settings might present unpredictable adverse effects; thus, we need to evaluate HLA mismatch–related rejection response in the long term.

In conclusion, local administration of MDSCs can alleviate the pathological development and progression of EAU by inhibiting oxidative stress and inflammation in innate immunity and suppressing Th1 and Th17 cells activation; this is possibly mediated by Treg cell induction in acquired immune pathways. To the best of our knowledge, this study is the first to evaluate the suppression of oxidative stress and inflammatory progression through the local delivery of MDSCs in EAU. This study provides supportive data for clinical trials using MDSCs as the new therapeutic agent and delivery system for various autoimmune ocular diseases.

## Supplementary Material

Supplement 1

Supplement 2
