# Supplementary material for: Local Myeloid-Derived Suppressor Cells Impair Progression of Experimental Autoimmune Uveitis by Alleviating Oxidative Stress and Inflammation
Source: Invest Ophthalmol Vis Sci. 2023 Oct 25;64(13):39. doi: 10.1167/iovs.64.13.39 (PMC10615146; doi:10.1167/iovs.64.13.39)
Supplement: Supplement 1 [file iovs-64-13-39_s001.pdf]

Supplementary Figure. 1

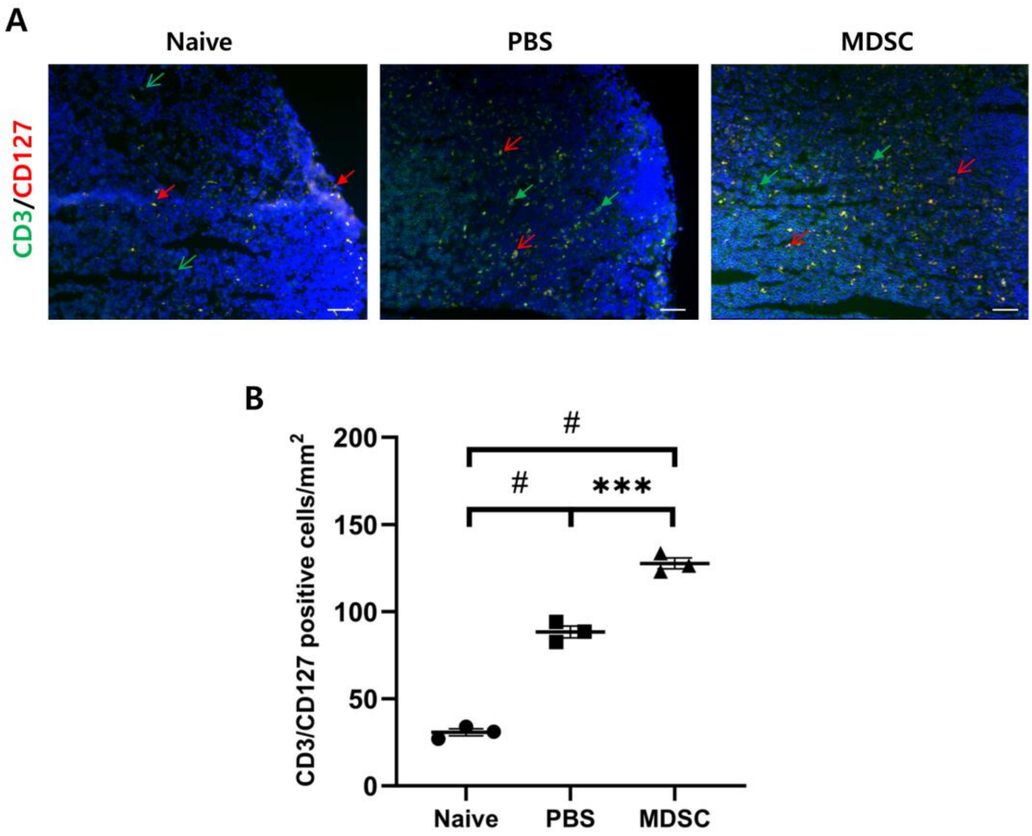

Supplementary Figure 1. Assessment of CD3+CD127+ Treg in draining lymph nodes at 3 weeks post-sensitization.

(A) Representative CD3+ CD127+ Treg labeling assay images of the draining lymph node cross-section each group. CD3 (green arrow, Cat# 100210, Alexa Fluor® 488, Biolegend, San Diego, CA, USA); CD127 (red arrow, Cat# sc-514445, AF 594, Santa Cruz Biotechnology, Dallas, TX, USA); counterstained with DAPI (blue). magnification 200×, scale bar 50μm. (B) Quantitative analysis graph of CD3+CD127+ positive Treg cells. MDSCs treatment mice showed increased populations of CD3+CD127+ Treg on the draining lymph node compared with the PBS group. The quantification of the image was analyzed by calculating the number of positive cell counts using the software Image J, as previously described.[36] Different three sections from 3 or more independent mice were randomly selected for counting blinded samples, and the average was calculated. Data were presented as average ± SD. *** $p < .001$, # $p < .0001$.

Supplementary Figure. 2

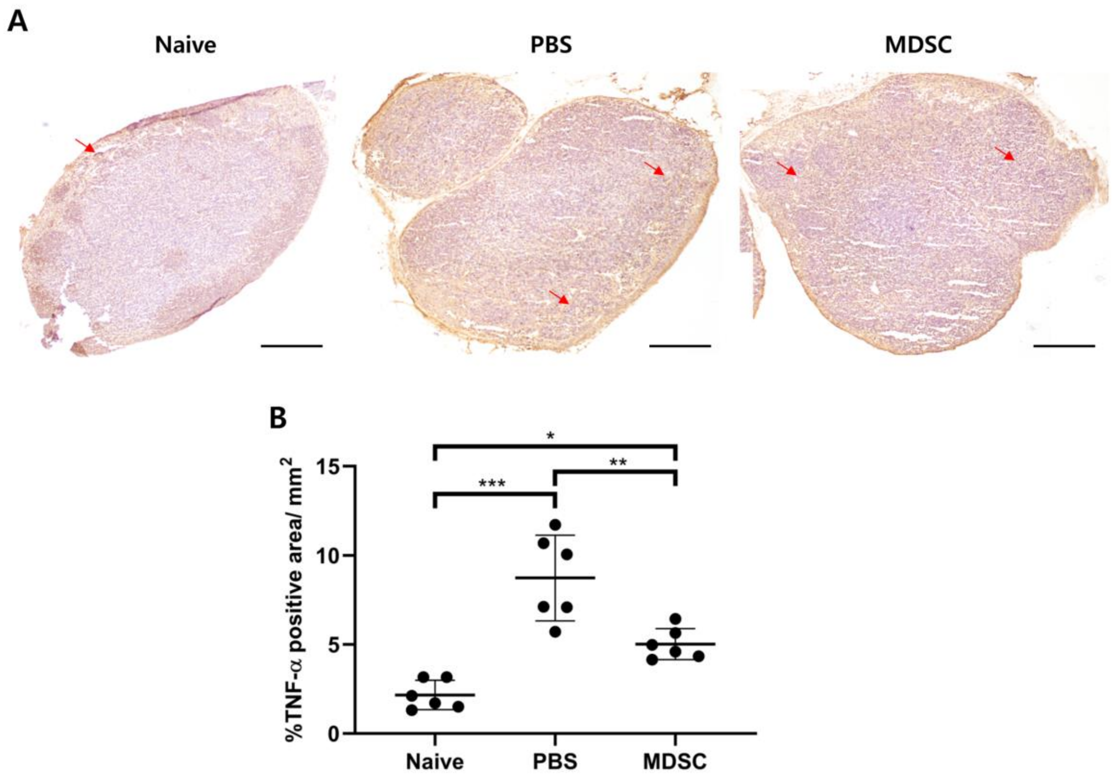

Supplementary Figure 2. Immunohistochemical staining of TNF-α cytokines in draining lymph nodes of EAU

(A) The histological-section images of draining lymph nodes showing immunohisto-chemical staining of TNF-α (red arrow for brown staining, Cat#sc-52746, Santa Cruz Biotechnology, TX, USA). magnification 200×, stitched images, scale bar 200μm. (B) Quantification graph for TNF-α cytokine positive area (%). MDSC groups presented the significantly suppressed expression of TNF-α cytokines in the lymph node, compared to the PBS group. The quantification of cytokine staining area was confirmed by calculating with the software Image J, as previously described.[36] Different 6 sections from over 3 mice were selected for the image analysis. Data were presented as average ± SD. * $p < .05$, ** $p < .01$, *** $p < .001$.

Supplementary Figure. 3

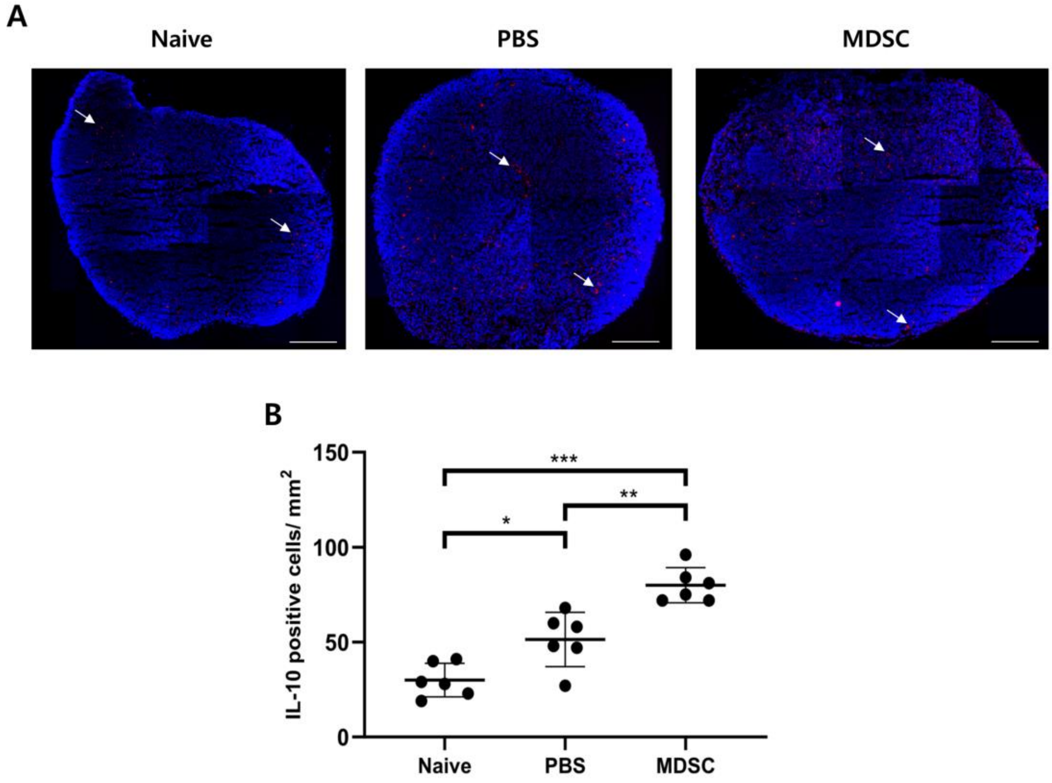

Supplementary Figure 3. Quantitative assay with immunofluorescence staining to IL-10 expression cells in draining lymph nodes of EAU.

(A) Representative IL-10 positive cell (white arrow, red staining, cat# 505008, PE, Biolegend, San Diego, CA, USA) images of the cross-section in draining lymph nodes of EAU each group. magnification 200×, stitched images, scale bar 200μm. (B) Quantification graph for IL-10 cytokine positive cells. MDSC groups showed increased population of IL-10 positive cells in draining lymph node, compared to the PBS group. The quantification of the field image was performed by calculating the number of positive cell counts using the Image J software, as previously described.[36] Different 6 sections from over 3 mice were selected for the image analysis. Data were presented as average ± SD. * $p < .05$, ** $p < .01$, *** $p < .001$.

Supplementary Figure. 4

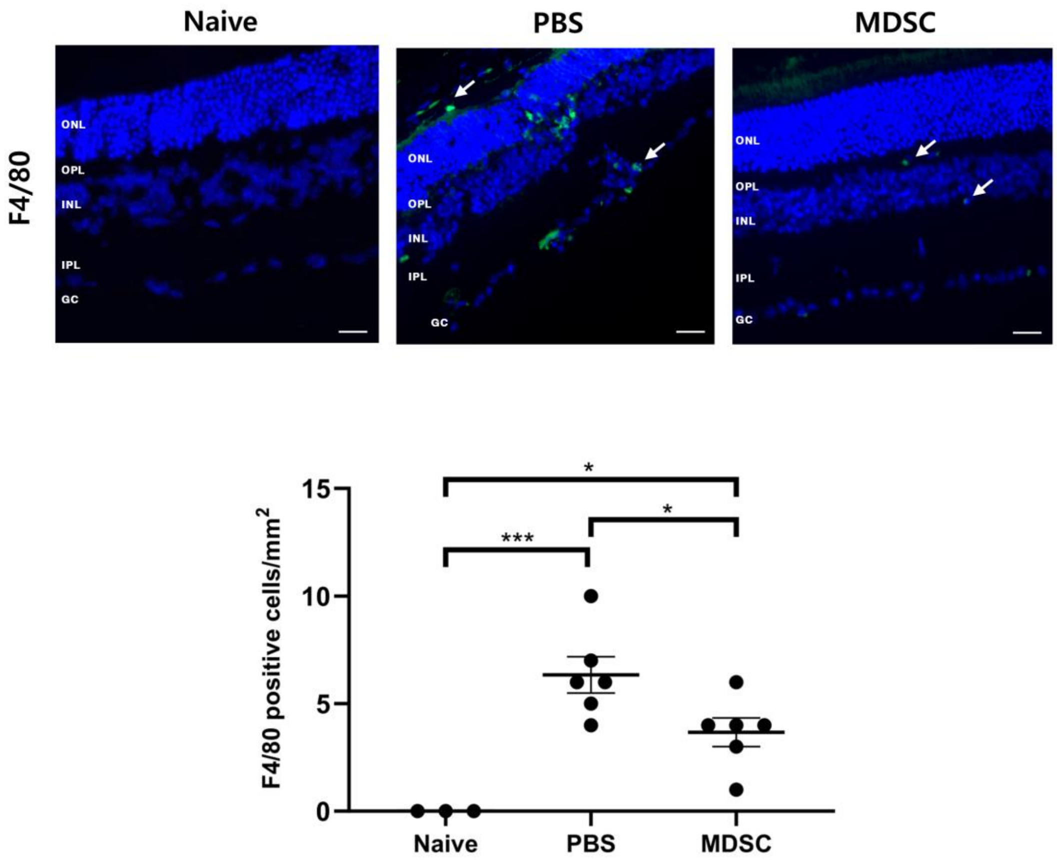

Supplementary Figure 4. Infiltration of F4/80+ cells in retinal inflammation of uveitis. (A) Representative infiltrated F4/80+ cells (white arrow for green staining, F4/80; Cat# 123120, Alexa Fluor® 488, Biolegend, San Diego, CA, USA) images of the retinal cross-section of each group, counterstained with DAPI (blue). magnification 200×, scale bar 50µm. (B) Quantitative analysis graph of F4/80+ positive macrophages. MDSCs treatment mice showed decreased populations of infiltrated F4/80 positive cells on the retina compared with the PBS group. The quantification of the image was analyzed by calculating the number of positive cell counts using the software Image J, as previously described.[36] Different 3 sections from 3 or more independent mice were randomly selected for counting and the average was calculated. Data were presented as average ± SD. * $p < .05$, *** $p < .001$.
