# Supplementary material for: Local Myeloid-Derived Suppressor Cells Impair Progression of Experimental Autoimmune Uveitis by Alleviating Oxidative Stress and Inflammation
Source: Invest Ophthalmol Vis Sci. 2023 Oct 25;64(13):39. doi: 10.1167/iovs.64.13.39 (PMC10615146; doi:10.1167/iovs.64.13.39)
Supplement: Supplement 2 [file iovs-64-13-39_s002.pdf]

see supplementary methods

1. Isolation of mononuclear cells (MNC) from human umbilical cord blood (UCB)

Human UCB was provided from the Catholic Hematopoietic Stem Cell Bank after written informed consent given by normal full-term pregnant women. For isolation of MNCs, 10% pentastarch was added as much as a quarter of the weight of UCB and gently mixed. The mixture was transferred into 50 mL conical tubes and centrifuged at 500 rpm for 20 min. After centrifugation, serum and buffy coat was carefully transferred on the top of the equal volume of Ficoll-Paque PLUS density gradient media (GE Healthcare, Cat.No.17-1440-03) and centrifuged at 2500 rpm for 20 min. Collect the buffy coat and perform serial washing centrifuge at 1800 rpm for 15min and 1600 rpm for 10 min using RPMI-1640 serum free medium (LONZA, Cat.No.12-702F). Finally, CB-derived MNC were storage concentration at $2x10^7$cells/vial and cryopreserved until use.

2. Culture of hUCB-MDSCs

All experimental procedures using human cord blood derivatives, including hUCB-MDSCs, were conducted under guidelines approved by Korea National Institute for bioethics policy (IRB no. P01-202010-31-008). hUCB-MDSCs were isolated and cultured according to a previously described method.[29] Briefly , CD34+ cells (Miltenyi Biotec, Bergisch Gladbach, Germany) were isolated and cultured in 48-well plates at 1×10^5 cells per well with 1 mL Iscove's Modified Dulbecco's Medium containing 10% heat-inactivated Fetal Bovine Serum (Gibco, Thermo Fisher Scientific, Waltham, MA, USA), 10% penicillin–streptomycin (100 U/mL; Lonza, Walkersville, MD), 2 mM L-glutamine (Lonza), 100 ng/mL recombinant human granulocyte-macrophage colony-stimulating hormone (CSF), and 50 ng/mL recombinant human Stem Cell Factor (SCF) (Peprotech, Rocky Hill, NJ, USA). After incubation for 7 days, the medium was changed every 7 days, and the cells were cultured for 3 weeks using the method described above. From weeks 4 to 6, the cells were cultured at a higher density (5×10^5 cells/well).

3. Preparation of hUCB-MDSCs administration

We thaw frozen cells vials in a 37°C water bath, viable hUCB-MDSCs were counted with trypan blue ($1x10^6$ cells/mice), washed with RPMI-1640 serum-free medium at 2 times (LONZA, Cat.No.12-702F) after centrifuging 1600 rpm for 10 min. Finally, total volume was prepared to be $1x10^6$ cells/10ul per mouse, temporarily stored on ice, and then rapidly used for administration.
